# Non-secretory Multiple Myeloma: A Report of a Rare Case

**DOI:** 10.7759/cureus.83759

**Published:** 2025-05-08

**Authors:** Jacob T Harris, Alexa Viniotis, John Bibb

**Affiliations:** 1 Internal Medicine, HonorHealth, Scottsdale, USA; 2 Hematology/Oncology, HonorHealth, Scottsdale, USA

**Keywords:** diagnosis of multiple myeloma, multiple myeloma, multiple myeloma treatment, newly diagnosed multiple myeloma, non-secretory multiple myeloma

## Abstract

Multiple myeloma (MM) is a rare blood cancer. Non-measurable MM (NMMM) is a category designation consisting of both non-secretory MM (NSMM) and oligo-secretory MM (OSMM), both of which have negative findings on serum protein electrophoresis and urine protein electrophoresis testing. NSMM and OSMM are infrequent subtypes. This report describes the case of a 67-year-old female patient without significant past medical history who presented with back pain, nausea, fatigue, and mild confusion. Labs were significant for severe hypercalcemia. Imaging demonstrated osteopenia and multiple lytic lesions throughout her spine. Serum protein electrophoresis and immunofixation by electrophoresis were obtained but were inconclusive. Bone marrow biopsy was therefore obtained to help clarify the diagnosis. Workup ultimately revealed a new diagnosis of kappa light chain-restricted NSMM. It is beneficial for the clinician to review the classic features of general MM in such a case, and they are also worth reviewing, as it may be better characterized as OSMM. Recognizing the rare features of NSMM and OSMM is relevant since these are entities not commonly encountered and can appear as classic MM in the initial workup. The care of patients will continue to improve as the medical community continues to recognize the rare malignancy of MM and its variant forms of NMMM, specifically NSMM and OSMM.

## Introduction

Multiple myeloma (MM) is a rare blood cancer that accounts for 1-2% of malignancies [1]. It is associated with bone pain or lytic lesions, monoclonal protein in the serum or urine, anemia, acute kidney failure, and hypercalcemia. It is important to note that MM, smoldering MM (SMM), and monoclonal gammopathy of undetermined significance (MGUS) are all entities that are part of a spectrum and are therefore not considered distinct diseases [1]. 

Non-measurable MM (NMMM) is a category designation consisting of non-secretory MM (NSMM) and oligo-secretory MM (OSMM). Both NSMM and OSMM have negative findings on serum protein electrophoresis (SPEP) and urine protein electrophoresis (UPEP) testing. NSMM, also referred to as true NSMM, is a rare subtype that makes up 3% of all MM, and in which SPEP and UPEP detect no monoclonal proteins, and serum free light chain (FLC) ratios are abnormal [1]. Immunofixation is a more advanced test that can sometimes show positive monoclonal proteins even if SPEP and UPEP are negative. When this occurs, the disease is considered OSMM. When even the immunofixation is negative, the disease is considered NSMM. 

This case report describes a patient with new concern for NSMM who initially presented with compression fractures and later hypercalcemia. Further discussion is provided to investigate OSMM as a possible alternative diagnosis. Such diagnoses are not regularly encountered by many clinicians, which makes their workup, diagnosis, and natural history valuable to review.

## Case presentation

A 67-year-old female presented to the hospital with nausea, vomiting, fatigue, anorexia, mild confusion, word finding difficulty, subjectively worsening depression, and increased back pain. Months prior to her current admission, she sustained an L4-L5 compression fracture after a ground-level fall. Initially, after the fall, she remained physically active. However, as her current nausea, fatigue, confusion, and back pain worsened, she became almost non-ambulatory. 

**Figure 1:**
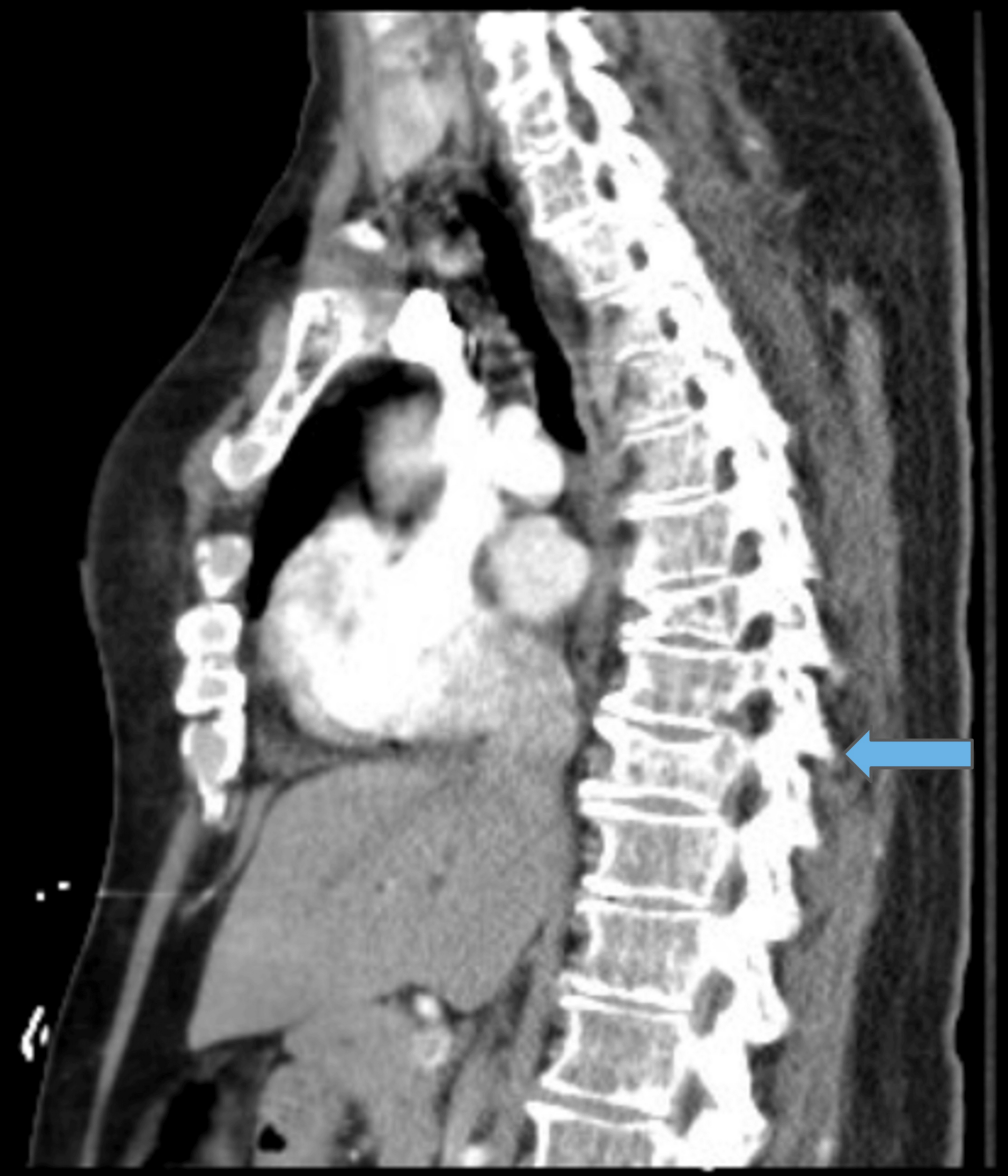
CT chest (sagittal view) demonstrating moderate compression deformities in the thoracic spine (blue arrow).

**Figure 2:**
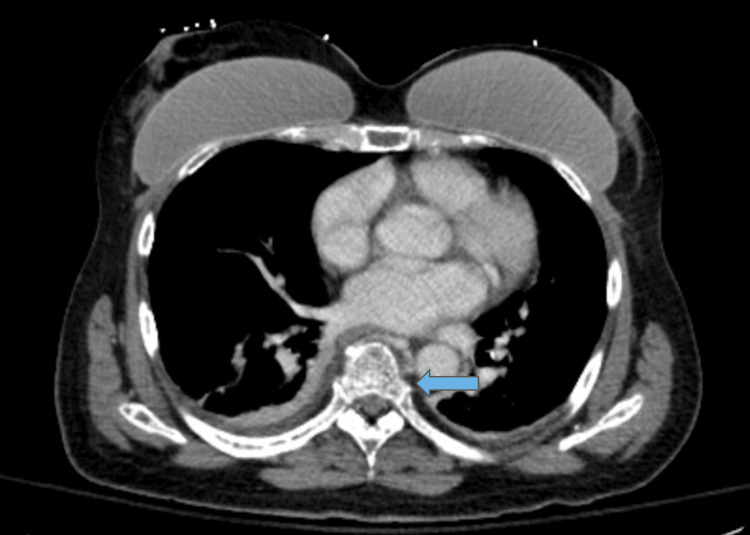
CT chest (axial view) demonstrating generalized osteopenia and lytic lesions in the thoracic spine likely due to multiple myeloma, less likely metastatic disease (blue arrow).

Vital signs were unremarkable. Physical exam showed no back tenderness, a somewhat guarded abdomen that was nontender and nondistended, some wordfinding difficulty, but otherwise unremarkable neurologic, cardiac, and pulmonary exam. CT chest/abdomen/pelvis showed generalized osteopenia with multiple lytic lesions and moderate compression deformities of the thoracic spine, as seen in Figures 1, 2. 

The patient's calcium was elevated at 15.7 mg/dL (ref 8.5-10.3 mg/dL), with ionized calcium >2.2 mmol/L (reference 1.03-1.23 mmol/L). Her hemoglobin was normal. For her severe hypercalcemia, urgent treatment was initiated with IV fluids, calcitonin, and zoledronic acid. A bone marrow biopsy was obtained and demonstrated plasma cell myeloma (cellularity: average 70%; plasma cells: 75%) with background bone marrow demonstrating reduced trilineage hematopoiesis and moderate reticulin fibrosis but no increase in the number of blasts (<1%). The biopsy also showed a population of monoclonal kappa light chain-restricted plasma cells positive for CD38 and CD138, negative for CD19, CD20, CD56, and CD117, and no amyloid deposition on Congo red stain. 

UPEP was inconclusive. SPEP showed no monoclonal proteins. The 24-hour urine protein was 770 mg/day (reference <137). In the outpatient setting after discharge, repeat SPEP again showed no monoclonal proteins. Immunofixation by electrophoresis (IFE) showed IgM was 9 mg/dL (reference 48-271), IgG was 535 mg/dL (reference 694-1618), and IgA was 23 mg/dL (reference 81-463). Free kappa light chain was found to be 13.95 mg/L (reference 3.3-19.4), free lambda light chain was 3.2 mg/L (reference 5.71-26.3), and free kappa/lambda ratio was 4.36 (reference 0.26-1.65). Beta-2-microglobulin was 2.5 mg/L (reference <3.0). Cytogenetics and myeloma fluorescence in situ hybridization (FISH) were negative. 

The patient was formally diagnosed with kappa light chain-restricted NSMM International Staging System (ISS) stage II and was initiated on treatment with daratumumab split dosing of 500 mg and 1000 mg, lenalidomide split dosing of 10 mg and 25 mg, bortezomib 1.3 mg/m2, and dexamethasone. PET-CT after initiation of treatment showed widespread osteolytic lesions throughout the axial and proximal appendicular skeleton, with other changes compatible with partial treatment response. The patient later underwent an autologous hematopoietic stem cell transplant. Due to the determined non-secretory nature of her multiple myeloma, the patient's disease was decided to be followed radiographically and with bone marrow biopsies rather than with SPEP, FLC, or 24-hour UPEP.

## Discussion

MM accounts for 1-2% of all malignancies and has various malignant and premalignant entities with which it is associated. The diagnosis of MM is based on the International Myeloma Working Group, which outlines two criteria. The first criterion is fulfilled with either clonal bone marrow plasma cells ≥10% or biopsy-proven bony or extramedullary plasmacytoma. The second criterion is fulfilled with either evidence of end-organ damage related to the underlying plasma cell proliferation disorder or a biomarker of malignancy. Evidences of end-organ damage include hypercalcemia (>0.25 mmol/L higher than the upper limit of normal or >2.75 mmol/L), renal insufficiency (creatinine clearance <40 mL/minute or serum creatinine >2 mg/dL), anemia (hemoglobin >2 g/dL below the lower limit of normal or hemoglobin <10 g/dL), or bone lesions (one or more osteolytic lesions on imaging) [2]. These clinical and laboratory findings were seen in the patient presented in the current case. The cognitive abnormalities are attributed to hypercalcemia-related encephalopathy, though they are sometimes mistaken for neurologic or mood pathologies. Biomarkers of malignancy include clonal bone marrow plasma cell percentage ≥60%, involved:uninvolved serum FLC ratio ≥100, or >1 focal lesion on MRI studies [2]. In simple terms, a patient with MM will typically present with hypercalcemia, renal function impairment, anemia, and bone pain (commonly referred to as the CRAB (calcium elevation, renal insufficiency, anemia, and bone abnormalities) mnemonic).

SMM and MGUS have different diagnostic criteria. SMM can be diagnosed if (i) serum monoclonal protein ≥30 g/L or urinary monoclonal protein ≥500 mg per 24 hours or clonal bone marrow plasma cells 10-59%, and (ii) there is an absence of myeloma-defining events or amyloidosis. The diagnosis of MGUS can be confirmed if serum monoclonal protein is <30 g/L, bone marrow plasma cells are <10%, and there is an absence of myeloma-defining events or amyloidosis [2].

Variants of MM are subdivided based on their immunoglobulins: IgG, IgA, IgD, IgM, kappa light chain, and lambda light chain. One analysis described each subtype’s prevalence and showed the negative immunoglobulin variant to have a prevalence of 6.5% [3]. This includes both OSMM and NSMM. OSMM is characterized by having serum M protein <1 g/dL and urine M protein <200 mg/24 hours. NSMM is characterized by having normal serum and urine immunofixation and normal serum FLC ratio [1].

The distinction between OSMM and NSMM within the broader category of NMMM is worth further discussion. A diagnosis of NSMM in the presented case may be questioned based on the UPEP, 24-hour urine protein, immunofixation, and serum FLC ratio results. All of these tests are used to diagnose forms of MM. SPEP and UPEP are techniques that separate proteins based on their physical properties and identify monoclonal proteins (e.g., Bence Jones proteins) [3]. Immunofixation is a more sensitive technique than SPEP and UPEP and is often utilized to identify the specific monoclonal proteins found in SPEP and UPEP testing [1,5]. However, in cases of NMMM, immunofixation can be used to identify monoclonal proteins that SPEP and UPEP did not identify, as in the present case. Finally, FLC testing measures the levels of free kappa and lambda light chains in the blood [4]. This is also a highly sensitive test that can detect proteins missed by SPEP and UPEP.

Some may argue that OSNM is the more appropriate diagnosis, rather than NSMM, for the patient in the current case. Some clarification is therefore needed. While UPEP identifies monoclonal proteins which are indicative of conditions like MM, a 24-hour urine protein level is a separate test that measures the overall proteinuria of a patient [3]. The causes of the proteinuria seen on this test are variable and can include MM as well as glomerular diseases and tubular disorders. Therefore, for the current patient with inconclusive UPEP and a 24-hour urine protein of 770 mg/day, NSMM could theoretically still be a possible diagnosis. However, our patient also had immunofixation results positive for monoclonal kappa light chains, and her serum FLC ratio was abnormal. These findings may potentially be used to argue for an OSMM diagnosis instead of true NSMM. Furthermore, immunophenotypic markers of CD38+, CD138+, and CD56- may be more indicative of OSMM than NSMM. Interestingly, the patient in the present case was diagnosed with kappa light chain restricted NSMM ISS stage II. This was done under the assumption that NSMM is defined as a subtype of MM where no monoclonal protein is detected in the serum or urine using conventional methods. This demonstrates the lack of clarity in diagnostic criteria that has already been noted in the literature [1]. On the one hand, NSMM is defined as having no monoclonal protein in conventional methods (i.e., SPEP and UPEP), which is in line with this patient being diagnosed with NSMM. On the other hand, newer and more sensitive diagnostic methods that may not be classified as conventional based on available NSMM diagnostic criteria (i.e., immunofixation) can demonstrate monoclonal protein, which would support this patient being diagnosed alternatively with OSMM. The newer and more sensitive technologies of immunofixation and FLC present a dilemma on how to characterize/label certain diseases that don’t fit clearly into current diagnostic criteria.

In general, the treatment for NSMM is the same as for MM. This is because although NSMM has undetectable monoclonal proteins, the disease pathophysiology is comparable to MM with detectable monoclonal proteins. That is, it can have similar organ damage or disease burden. Induction therapy typically includes a monoclonal antibody, a proteasome inhibitor, an immunomodulator, and a corticosteroid [4,5]. Patients who are eligible are also recommended to pursue autologous stem cell transplantation, as this has shown favorable outcomes with improved progression-free survival of >20 months [6]. It is also common to utilize lenalidomide (immunomodulator) as maintenance therapy to prolong remission.

In many cases of NSMM, disease progression is monitored with imaging and bone marrow biopsies since SPEP, UPEP, and FLC are inadequate methods because no proteins are produced [1]. However, these methods may have benefits when monitoring OSMM. It is therefore unclear what specific monitoring modalities would be of most benefit for the patient presented in this report. Other reported monitoring methods for NSMM include circulating plasma cells [7], cell-free DNA [7], and, as described in a recent study by Ikeda et al., soluble B-cell maturation antigen [8]. Considering all these methods, monitoring for the disease remains an individualized matter based on measurable disease markers. With adequate treatment, NSMM with undetectable IgM appears to have a slightly better prognosis compared to traditional secretory MM (progression-free survival of 28.6 months and 23.0 months, respectively) [9,10].

## Conclusions

NSMM and OSMM are uncommon subtypes of a rare malignancy. Diagnosis of MM follows established criteria involving bone marrow biopsy and abnormalities in calcium, renal function, hemoglobin, bone imaging, and biomarkers. Monitoring NSMM often requires bone marrow biopsies and imaging since traditional blood or urine-based markers such as SPEP, UPEP, and serum FLC are not being produced and are not measurable like in typical MM. However, these modalities can be beneficial in monitoring OSMM. The different sensitivities of diagnostic tests can guide clinicians to proper diagnoses, but uncertainty can still persist, as seen in the patient presented in this report. Clinicians must continue to have a high index of suspicion for MM since variants that do not follow classical presentations (e.g., NSMM) are often overlooked, and anchoring bias can cause physicians to focus on alternative diagnoses. This will improve timely intervention and preserve the patient's quality of life. With increasingly more medical literature discussing such, perhaps revised definitions and diagnostic criteria for MM are needed.
